# Urinary peptides provide information about the risk of mortality across a spectrum of diseases and scenarios

**DOI:** 10.1186/s12967-023-04508-6

**Published:** 2023-09-24

**Authors:** Felix Keller, Joachim Beige, Justyna Siwy, Alexandre Mebazaa, Dewei An, Harald Mischak, Joost P. Schanstra, Marika Mokou, Paul Perco, Jan A. Staessen, Antonia Vlahou, Agnieszka Latosinska

**Affiliations:** 1 Department of Internal Medicine IV (Nephrology and Hypertension), Medical University Innsbruck, 6020 Innsbruck, Austria; 2 Martin-Luther-University Halle-Wittenberg, 06108 Halle (Saale), Germany; 3 Kuratorium for Dialysis and Transplantation, 04129 Leipzig, Germany; 4 Mosaiques Diagnostics GmbH, 30659 Hannover, Germany; 5 Department of Anaesthesiology and Critical Care, Hôpital Lariboisière, AP-HP, 75010 Paris, France; 6 Non-Profit Research Association Alliance for the Promotion of Preventive Medicine, 2800 Mechelen, Belgium; 7 Institute of Cardiovascular and Metabolic Disease, U1297, Institut National de la Santé et de la Recherche Médicale, 31432 Toulouse, France; 8 Université Toulouse III Paul-Sabatier, 31062 Toulouse, France; 9 Center of Systems Biology, Biomedical Research Foundation of the Academy of Athens, 11527 Athens, Greece

**Keywords:** Death, Mass spectrometry, Peptidomics, Personalised medicine, Urinary biomarkers

## Abstract

**Background:**

There is evidence of pre-established vulnerability in individuals that increases the risk of their progression to severe disease or death, although the mechanisms causing this are still not fully understood. Previous research has demonstrated that a urinary peptide classifier (COV50) predicts disease progression and death from SARS-CoV-2 at an early stage, indicating that the outcome prediction may be partly due to vulnerabilities that are already present. The aim of this study is to examine the ability of COV50 to predict future non-COVID-19-related mortality, and evaluate whether the pre-established vulnerability can be generic and explained on a molecular level by urinary peptides.

**Methods:**

Urinary proteomic data from 9193 patients (1719 patients sampled at intensive care unit (ICU) admission and 7474 patients with other diseases (non-ICU)) were extracted from the Human Urinary Proteome Database. The previously developed COV50 classifier, a urinary proteomics biomarker panel consisting of 50 peptides, was applied to all datasets. The association of COV50 scoring with mortality was evaluated.

**Results:**

In the ICU group, an increase in the COV50 score of one unit resulted in a 20% higher relative risk of death [adjusted HR 1.2 (95% CI 1.17–1.24)]. The same increase in COV50 in non-ICU patients resulted in a higher relative risk of 61% [adjusted HR 1.61 (95% CI 1.47–1.76)], consistent with adjusted meta-analytic HR estimate of 1.55 [95% CI 1.39–1.73]. The most notable and significant changes associated with future fatal events were reductions of specific collagen fragments, most of collagen alpha I (I).

**Conclusion:**

The COV50 classifier is predictive of death in the absence of SARS-CoV-2 infection, suggesting that it detects pre-existing vulnerability. This prediction is mainly based on collagen fragments, possibly reflecting disturbances in the integrity of the extracellular matrix. These data may serve as a basis for proteomics-guided intervention aiming towards manipulating/ improving collagen turnover, thereby reducing the risk of death.

**Supplementary Information:**

The online version contains supplementary material available at 10.1186/s12967-023-04508-6.

## Background

Pre-existing vulnerabilities play a key role in determining an individual’s risk for disease progression or death [1], highlighting the importance of considering these factors when managing diseases. Given the complexity of the disease-associated molecular mechanisms [2–5] and the factors impacting the outcome, recognizing and understanding the pre-established vulnerabilities can help identify high-risk individuals and tailor treatment strategies, ultimately improving outcome.

Among theories attempting to explain this phenomenon (e.g., in the context of trauma) [6], the “two-hit” model has evolved. According to this scenario, the stress response encompasses the physiological reaction to the initial injury (referred to as the “first hit”), followed by a reaction to the secondary insult/ intervention (known as the “second hit”) [6, 7]. The model is rooted in the fundamental idea that consecutive insults, which may not have significant effects individually, can result in a profound physiological response. This reaction can manifest in various biological systems and can be evaluated by measuring multiple parameters [8]. However, the molecular mechanisms responsible for the “two-hit” model are complex and not fully understood [6, 8]. In general, the “first hit” acts as a priming event that predisposes the patient to develop a systemic inflammatory syndrome, with a key feature being a leak of the endothelium. This initially manifests in a specific body region, but eventually affects multiple organs. Subsequently, a second insult can trigger an exaggerated inflammatory response, responsible for potentially life-threatening conditions such as multiple organ failure and multiple organ dysfunction syndrome [6, 8]. Understanding the vulnerability to the “second hit” can support minimising the impact of complications, potentially leading to a better outcome.

Recently, it has been suggested that SARS-CoV-2 infection could act as a “second hit”. SARS-CoV-2 is among the main conditions associated with collapsing glomerulopathy, acting as a “second hit” in susceptible patients with *APOL1* risk alleles, similar to human immunodeficiency virus and other viruses [9]. Another example involves complement-mediated disorder, which seems to be a predominant form of thrombotic microangiopathy associated with COVID-19. Considering the development of thrombotic microangiopathy following SARS-CoV-2 infection, it was suggested that the virus acted as a “secondary trigger”, revealing an underlying complement defect [10].

Previous biomarker research demonstrated the capability of a urinary peptide-based classifier (COV50) to predict disease progression and death from SARS-CoV-2 at the earliest possible date, i.e., upon the first positive indication of a SARS-CoV-2 infection [11, 12]. This assessment was based on the measurement of 50 specific urinary peptides, with the most prominent changes involving the reduction of peptides derived from collagen alpha 1(I), polymeric immunoglobulin receptor and CD99 antigen, and an increase in peptides derived from alpha-1-antitrypsin [13]. The ability to predict outcome very soon after infection suggests that the prediction may not be solely based on molecular events associated with SARS-CoV-2 infection, but at least in part due to pre-established vulnerability, resulting from a “first hit”. This would indicate that prediction of severe disease course may be feasible even before the infection. We hypothesised that this pre-established vulnerability can be generic (expanded to other indications) and COV50 could serve as a biomarker for detecting vulnerable subjects who are adversely affected by other clinical insults.

Hence, the present study aimed to examine the ability of COV50 to predict future non-COVID-19-related mortality in patients admitted to the intensive care unit (ICU) or having other diseases (non-ICU). If the hypothesis is confirmed, a significantly higher number of individuals in this vulnerable population (defined by a high COV50 score) should experience death compared to the population with a lower score.

## Methods

### Patients

ICU: Patients from the medical, surgical, or mixed ICUs at 14 university hospitals from the FROG-ICU study were included [14]. Inclusion criteria were mechanical ventilation or administration of vasoactive agents for at least 24 h. Exclusion criteria were age under 18, severe head injury with a Glasgow Coma Scale below 8, brain death or persistent vegetative state, pregnancy or breastfeeding, transplantation in the past 12 months, moribund status, and lack of social security coverage. All capillary electrophoresis coupled to mass spectrometry (CE-MS) datasets with a 1-year follow-up and information on relevant co-variables (age, body mass index (BMI), sex, blood pressure, estimated glomerular filtration rate (eGFR), presence of diabetes, kidney, cardiovascular disease, hypertension) were included in the present study without pre-selection.

Non-ICU: The assessment of COV50 in the non-ICU population was based on 7474 datasets from the Human Urinary Proteome Database [15, 16] with available information on age, sex, eGFR, blood pressure, BMI, presence of diabetes, kidney disease, cardiovascular disease, hypertension, and a follow-up data.

All datasets were obtained from previously published studies and fully anonymized. Ethical review and approval were waived for this study by the ethics committee of the Hannover Medical School, Germany (no. 3116-2016), as all data were fully anonymized. The number of subjects per study and patient characteristics are listed in Table 1 and Additional file 1.

**Table 1:** Descriptive statistics for the ICU and non-ICU samples analysed within this study

	Level/unit	ICU				Non-ICU			
		Overall	Death: no	Death: yes	p	Overall	Death: no	Death: yes	p
N		1719 (100%)	1139 (66,3%)	580 (33.7%)		7474 (100%)	6849 (91.6%)	625 (8.4%)	
Study	FROG	1719 (100%)	1139 (100%)	580 (100%)					< 0.001
	CAD Predictions					145 (1.9%)	50 (0.7%)	95 (15%)	
	CardioRen					116 (1.6%)	87 (1.3%)	29 (4.6%)	
	DIRECT					1,487 (20%)	1,448 (21%)	39 (6.2%)	
	EPOGH					914 (12%)	850 (12%)	64 (10%)	
	EU Priority					1769 (24%)	1756 (26%)	13 (2.1%)	
	GenScot					473 (6.3%)	417 (6.1%)	56 (9.0%)	
	Heart Failure					84 (1.1%)	67 (1.0%)	17 (2.7%)	
	Homage Fibrosis					354 (4.7%)	229 (3.3%)	125 (20%)	
	PersTIgAN					270 (3.6%)	265 (3.9%)	5 (0.8%)	
	Predictions					91 (1.2%)	85 (1.2%)	6 (1.0%)	
	PROPHET					462 (6.2%)	444 (6.5%)	18 (2.9%)	
	STOP IgAN					109 (1.5%)	107 (1.6%)	2 (0.3%)	
	Sun Makro					581 (7.8%)	556 (8.1%)	25 (4.0%)	
	TransBioBC					131 (1.8%)	117 (1.7%)	14 (2.2%)	
	UZ Gent					488 (6.5%)	371 (5.4%)	117 (19%)	
Age	[yrs]	62 (50, 73)	58 (46, 69)	70 (61, 78)	< 0.001	60 (48, 68)	59 (47, 66)	73 (66, 79)	< 0.001
Female	Yes	602 (35%)	414 (36%)	188 (32%)	0.11	2,857 (38%)	2,657 (39%)	200 (32%)	< 0.001
BMI	[kg/m^2^]	26.2 (22.9, 30.0)	26.2 (22.8, 29.9)	26.4 (23.1, 30.1)	0.6	27.5 (24.3, 31.2)	27.6 (24.3, 31.4)	26.9 (23.7, 30.1)	< 0.001
Systolic BP	[mmHg]	123 (109, 140)	124 (110, 140)	120 (107, 139)	0.01	132 (121, 145)	132 (121, 144)	138 (124, 153)	< 0.001
Diastolic BP	[mmHg]	64 (55, 75)	66 (56, 77)	60 (52, 70)	< 0.001	79 (72, 85)	79 (73, 85)	75 (67, 82)	< 0.001
Mean Arterial BP	[mmHg]	84 (74, 95)	86 (76, 96)	80 (71, 92)	< 0.001	97 (90, 104)	97 (90, 104)	97 (88, 105)	0.3
Hypertension	Yes	979 (57%)	602 (53%)	377 (65%)	< 0.001	3090 (41%)	2758 (40%)	332 (53%)	< 0.001
eGFR	[ml/min/1.73 m^2^]	87 (48, 127)	97 (57, 132)	67 (37, 107)	< 0.001	82 (59, 99)	84 (62, 100)	61 (37, 80)	< 0.001
Kidney Disease	Yes	716 (42%)	378 (33%)	338 (58%)	< 0.001	2212 (30%)	1898 (28%)	314 (50%)	< 0.001
Diabetes	Yes	280 (16%)	160 (14%)	120 (21%)	< 0.001	4101 (55%)	3938 (57%)	163 (26%)	< 0.001
Cardiovascular Disease	Yes	98 (5.7%)	49 (4.3%)	49 (8.4%)	< 0.001	1357 (18%)	983 (14%)	374 (60%)	< 0.001
COV50		1.17 (0.34, 1.83)	1.01 (0.11, 1.74)	1.45 (0.72, 1.97)	< 0.001	− 1.88 (− 2.33, − 1.27)	− 1.89 (− 2.34, − 1.30)	− 1.69 (− 2.26, − 0.94)	< 0.001
FU Duration	[month]	12.0 (2.0, 12.1)	12.0 (12.0, 12.5)	0.7 (0.3, 2.1)	< 0.001	47 (29, 67)	48 (29, 67)	38 (19, 62)	< 0.001

Categorical variables are described with absolute (N) and group-wise relative frequencies (%), continuous variables with median (IQR). P-values for group differences result from chi-squared homogeneity tests for categorical and for Wilcoxon rank sum test for continuous variables

*BMI* body mass index, *BP* blood pressure, *eGFR* estimated glomerular filtration rate, *FU* follow-up, *ICU* intensive care unit, *yrs* years

### Urinary proteome/peptidome data

The urinary proteome is well characterized and reference standards are available [17]. Urinary proteome analysis was conducted on urine samples collected at study inclusion and subsequently bio-banked until assayed. Detailed information on urine sample preparation, proteome analysis by CE-MS, data processing, and sequencing of the urinary peptides allowing for the identification of parental proteins is available in previous publications [11, 18–20] and described in detail in Additional file 2.

### Outcome

In the FROG-ICU study, information on vital status was collected 3, 6, and 12 months after ICU discharge, as previously described [21]. For the non-ICU patients, vital status and outcome were assessed as described in the respective original studies [18, 22–37].

### Statistics

As descriptive statistics for the ICU and non-ICU samples, shown in Table 1, median and 1^st^ and 3^rd^ quartile (IQR) were used for continuous variables and absolute (N) and relative frequencies (%) for categorical variables. Hypotheses of no differences in scale or distribution of patient characteristics between the death and non-death groups were tested with Wilcoxon–Mann–Whitney tests for continuous and with χ^2^-homogeneity tests for categorical variables.

**Fig. 1:**
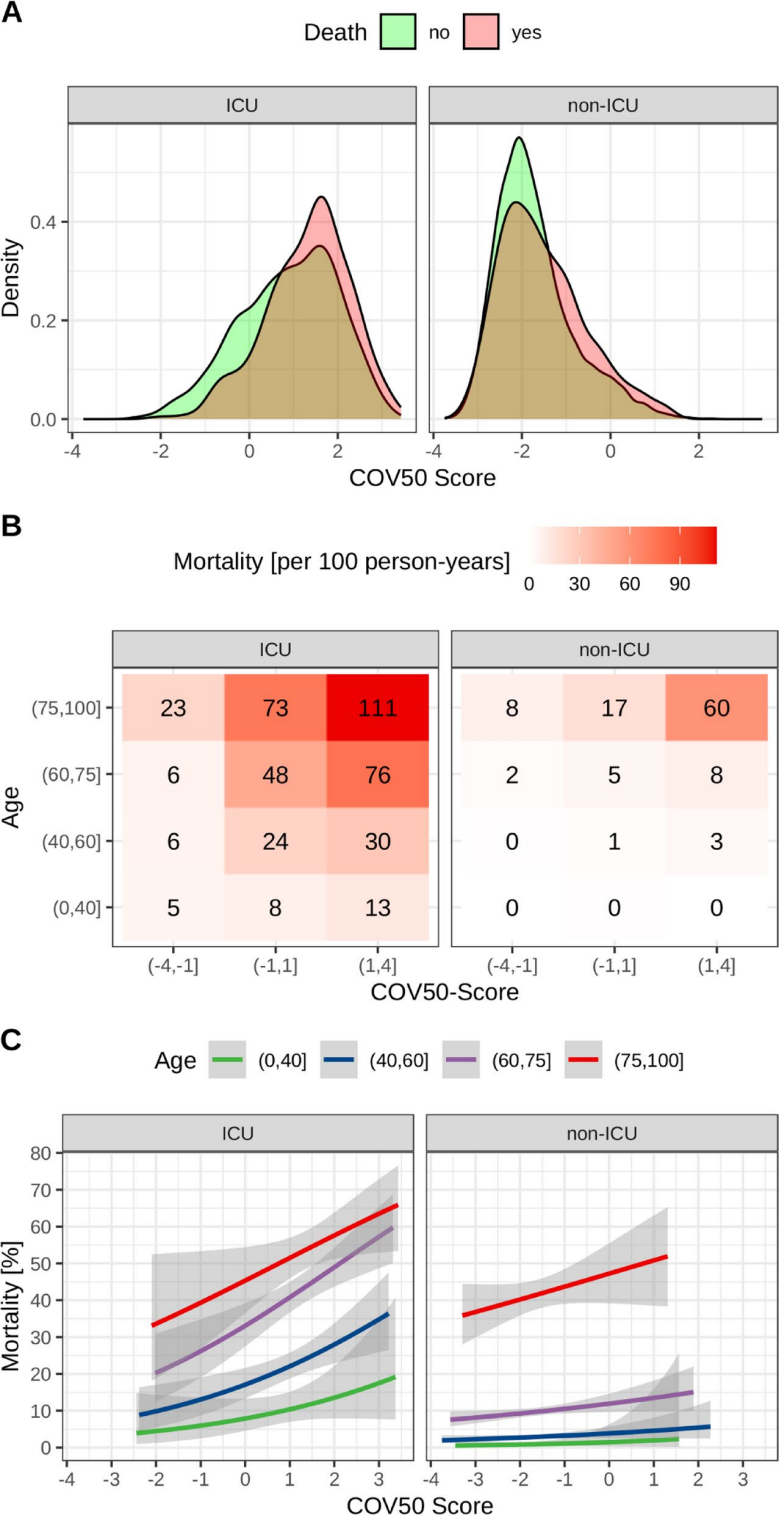
The distribution of COV50 scores. **A** Density of the COV50 distribution in ICU and non-ICU subjects. **B** Mortality per person-years for FROG and non-ICU cohorts given age and COV50. **C** Mortality as share [0–1] from a logistic regression for FROG and non-ICU cohorts given age and COV50 score

Kernel density estimates of the distribution of COV50 scores divided by ICU and mortality groups are depicted in Fig. 1A. Mortality per person-time, stratified by age and COV50 groups, as shown in Fig. 1B, was estimated as the ratio of the number of the deceased to the sum of all patients` observation times within each group, scaled to 100 person-years. Corresponding mortality probabilities with their 95% confidence intervals (CI) for each group, presented in Fig. 1C, were estimated through a logistic regression involving all 9193 patients.

**Fig. 2:**
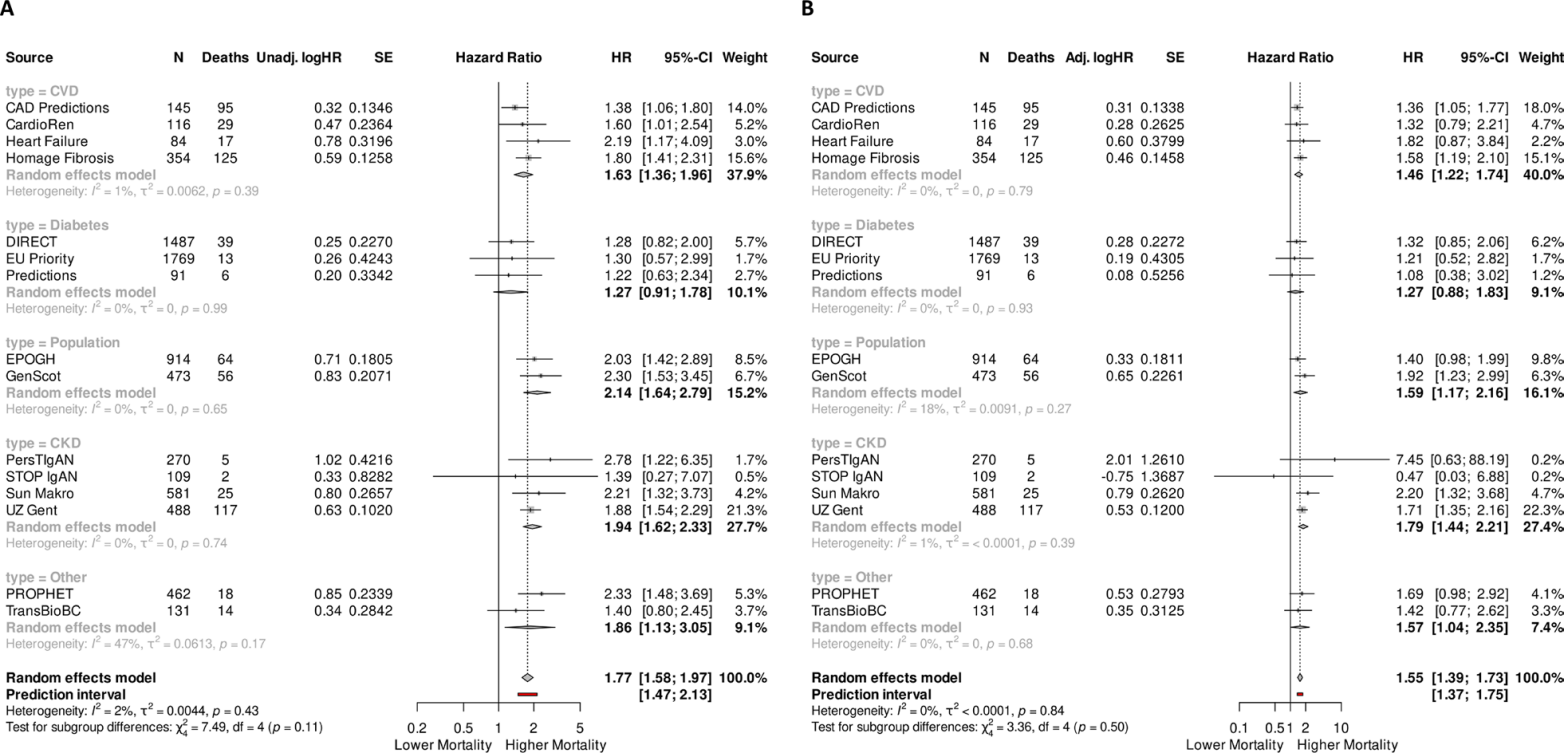
Random effects meta-analyses based on the log-HR and the standard errors from the separate cox regressions. **A** Unadjusted, **B** adjusted for sex, age, kidney function and BMI. The size of dot symbols is proportional to weight and weight is inverse proportional to HR standard error

For each study, separate unadjusted Cox regressions were conducted to analyse the effect of the COV50 score on experiencing death, as listed in Fig. 2A. In Fig. 2B, these models were additionally adjusted for age, female, log(BMI), mean arterial pressure (MAP) and log(eGFR). All regressors besides female and COV50 were normalized (mean 0, sd 1). The natural logarithms of the estimated hazard ratios (logHR) and their standard errors were combined in meta-analyses to determine the effect of the COV50 score on mortality. A random effects model was estimated after the assumption that all included studies are heterogeneous, i.e., coming from different populations. Study weights are based on the logHR estimates` uncertainty, namely their standard errors. Studies were categorized into more homogenous subgroups, and estimates for each subgroup were displayed in Fig. 2. Overall and group-wise between-study heterogeneity is presented with τ^2^ and assessed by Higgins & Thompson’s I^2^ statistic. χ^2^-Tests for heterogeneity and subgroup differences were based on Cochran’s Q. Random effects meta-analysis estimates were presented with 95% CIs and a 95% prediction interval for the overall effect. One Cox regression, stratified by study pooling all 9193 patients, was used as a benchmark to the meta-analytic approach. As displayed in Table 2, the model`s adjustment specification matched to the adjusted separate study regressions (Fig. 2B). To be comparable to the adjusted meta-analytic estimate, HRs for COV50 interacted with ICU and non-ICU, as well as for the above-mentioned non-ICU subgroups, were estimated. Standard errors were clustered on the study level for more robust inference and due to unobserved heterogeneity between studies. The models log-likelihood, associated Wald test and concordance were reported in Table 2. We allowed for a type 1 error of 5%, all hypotheses were two-sided. All analyses were carried out using R 4.2.2.

**Table 2:** Estimates from the pooled adjusted Cox regression

Effect	Group	(Non-ICU Subgroup)	HR	95% CI	p
Age			1.83	1.43, 2.35	< 0.001
Female			0.78	0.69, 0.87	< 0.001
log(BMI)			0.91	0.87, 0.94	< 0.001
MAP			0.88	0.84, 0.93	< 0.001
log(eGFR)			0.89	0.84, 0.95	< 0.001
COV50	ICU		1.2	1.17, 1.24	< 0.001
	Non-ICU		1.61	1.47, 1.76	< 0.001
		CVD	1.51	1.36, 1.68	< 0.001
		Diabetes	1.24	1.23, 1.25	< 0.001
		Population	1.68	1.31, 2.15	< 0.001
		CKD	1.82	1.70, 1.94	< 0.001
		Other	1.75	1.45, 2.11	< 0.001

All regressors besides Female and COV50 are were normalized to mean 0 and sd 1

n = 9193; N events = 1205;

statistic.wald = 7,096; p =  < 0.001;

c-index = 0.618; c-index SE = 0.013;

Log-likelihood = − 5959

*BMI* body mass index, *CI* Confidence Interval, *CKD* chronic kidney disease, *eGFR* estimated glomerular filtration rate, *HR* Hazard Ratio, *ICU* intensive care unit; *MAP* mean arterial pressure

## Results

First, we assessed the hypothesis that the COV50 classifier defines a vulnerable population at the molecular level, irrespective of SARS-CoV-2 infection. For that purpose, we examined datasets from subjects from the FROG-ICU study [21], as this study was more comparable to the CRIT-COV study (patients in ICU) and had available a large number of endpoints. We identified 1719 datasets to be included in this study, for which follow-up and information on relevant co-variables were available [38].

To further support our analysis, we also investigated whether COV50 could predict mortality in subjects outside the ICU. Studies with more than 50 individuals and available follow-up and information on relevant co-variables were selected from the Human Urinary Proteome Database [15, 16].

Demographic information on the subjects included in the study is presented in Table 1, separated into ICU and non-ICU groups, as well as by death status. More detailed information is provided in Additional file 1. Among the risk factors for death, we found significant differences at the aggregate level in both, the ICU and the non-ICU subgroups, as expected. The median COV50 score is significantly higher (p < 0.001) in patients who experienced death during the observation period, as also displayed in its distributions in Fig. 1A.

Considering that age is a crucial risk factor for mortality, we investigated the relationship between COV50 and mortality across different age groups. The results are illustrated in Fig. 1B, C. Panel B depicts mortality in person-time in COV50 groups, whereas panel C relates mortality as a percentage along the continuum of COV50 scores. In both subgroups, an increase in COV50 accompanies higher mortality, with this effect being more pronounced among older individuals.

The crude HRs in Fig. 2A for all studies generally indicate an association of a higher relative risk of death with increasing COV50 scores, with all but 5 studies showing a significantly elevated relative risk. In the meta-analysis, the combined HR estimate for all subgroups, except the diabetes-related studies, significantly differs from 1, as evident from the 95% CIs. Overall, the adjustment for risk factors reduced the COV50 HR estimates, in line with expectations, as adjustment typically improves comparability by accounting for observed between study heterogeneity at the patient level. However, in studies with low numbers of events (particularly PersTIgAN, STOPIgAN), variance increased substantially with the adjustment. The estimates from the meta-analysis resulted in an unadjusted HR of 1.77 [95% CI 1.58–1.97] and an adjusted HR of 1.55 [95% CI 1.39–1.73]. Although displaying a trend within and between the subgroups, neither heterogeneity nor subgroup differences were found to be statistically significant (Fig. 2).

The subgroup HR estimates derived from the adjusted meta-analysis in Fig. 2B are robust, as they closely align with the corresponding estimates from the pooled adjusted Cox regression shown in Table 2. Within the ICU group, an increase in the COV50 score of one unit results (on average) in a 20% higher relative risk of death [adj. HR 1.2 (95% CI 1.17–1.24)]. As the absolute risk of death is considerably lower in non-ICU patients, the same increase in COV50 in non-ICU patients results in a higher relative risk of 61% [adj. HR 1.61 (95% CI 1.47–1.76)]. These findings align well with the adjusted HR estimate of 1.55 [95% CI 1.39–1.73] obtained from the meta-analysis.

COV50 is a composite score based on 50 distinct urinary peptides. To examine which of these 50 peptides served as individual predictors of death in the cohorts investigated (ICU, non-ICU), we compared the distribution of the 50 peptides in the datasets from survivors with those from subjects that died. The results of this analysis are shown in Table 3. A high degree of concordance was observed when comparing the peptides regulation trend in the context of COVID-19, death in or after ICU, or death without ICU stay. The association of single peptides with the mortality was also supported by the Cox regression analysis. The most notable and significant changes associated with future fatal events are the reductions in specific collagen fragments, with most of them derived from collagen alpha I(I).

**Table 3:**
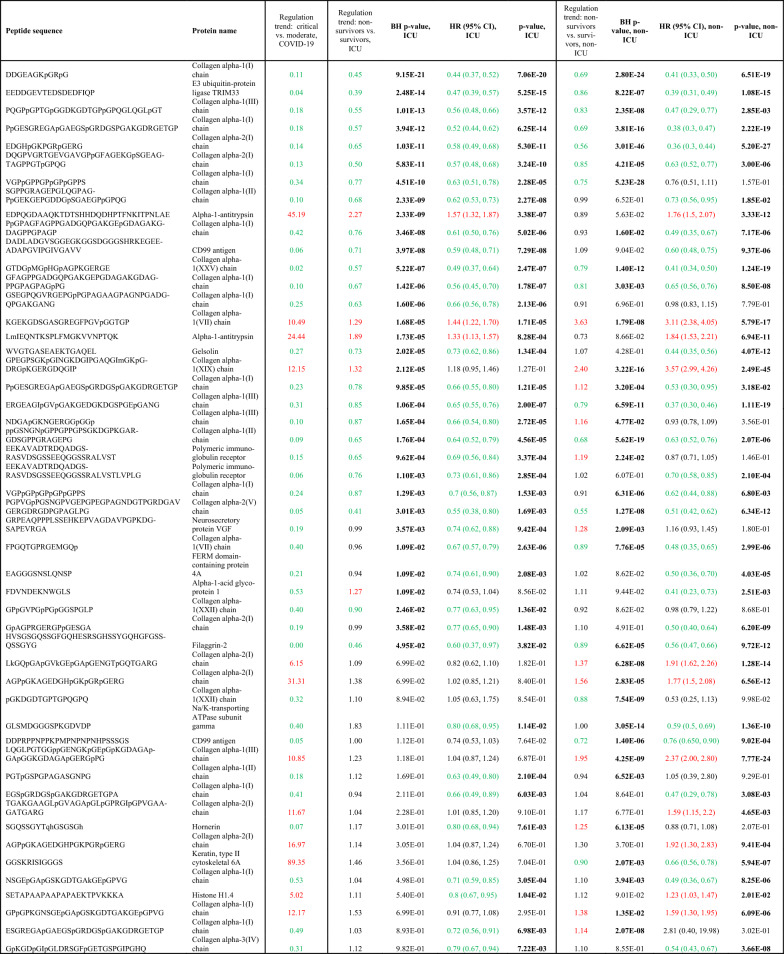
Urinary peptides included in the COV50 classifier

List of 50 urinary peptides included in the COV50 classifier and their respective regulation trend in investigated cohorts (ICU and non-ICU), and corresponding HRs for predicting mortality. P-values below 0.05 are marked in bold. Peptides with an increased abundance in the case vs. the control group (> 1.1) are marked in red, while those with decreased abundance (< 0.91) are marked in green. The regulation trend was calculated by dividing average abundances in the individual case vs. the control group

*BH* Benjamini-Hohberg, *CI *confidence interval, *HR* hazard ratio, *ICU* intensive care unit

In the ICU subjects, 33 out of the 50 peptides were found to be significantly associated with future death, with 29 having a regulation trend > 1.1 or < 0.91. Among the latter, 28 exhibited a regulation trend in a similar direction as for critical/lethal COVID-19. However, one peptide from alpha-1-acid glycoprotein 1 had an opposing regulation. Upon investigating the most prominent peptides derived from collagen, all significant changes were concordant between death in COVID-19 or ICU. In the non-ICU subjects, 34 of the 50 peptides were significantly associated with future death, with 29 having a regulation trend > 1.1 or < 0.91. Of these 29 peptides, 22 showed a regulation concordant with the one in critical/lethal COVID-19, while 7 peptides changed in an opposing direction. The latter group includes peptides derived from polymeric immunoglobulin receptor, neurosecretory protein VGF, keratin, type II, hornerin, collagen alpha-1(I), and collagen alpha-1(III). The most prominent difference in comparison to the distribution in COVID-19 patients was observed for peptides derived from CD99 antigen and polymeric immunoglobulin receptor. While a consistent trend and significant reduction of multiple CD99 antigen and polymeric immunoglobulin receptor peptides was associated with severe disease and mortality in critical COVID-19 patients, this distribution was less evident or not observed for polymeric immunoglobulin receptor in the non-ICU population and for CD99 antigen in all cohorts not infected with SARS-CoV-2.

## Discussion

This study is the first to investigate a peptide-based classifier, COV50, and specific urinary peptides in a large and diverse population of patients both inside and outside the ICU. The data demonstrate that COV50 not only predicts an unfavourable outcome of a COVID-19 episode but also appears to identify “vulnerable subjects” who are likely at substantially higher risk of severe or lethal COVID-19. This vulnerability also seems relevant in other clinical situations (e.g., non-SARS-CoV-2 infections), including those that lead to ICU admission, thereby increasing the risk of death in various pathological conditions. This is likely related to the fact that urinary peptides reflect local and systemic changes. It has been proposed that approximately 70% of urinary proteins under normal physiological circumstances are derived from the kidney and the urinary tract. The remaining 30% originates from other organs and is released into the bloodstream [39]. Although some of the peptides contained in COV50 have been previously identified in plasma [40, 41], the origin of specific naturally occurring urinary peptides cannot be predicted with high certainty. The most prominent and consistent findings are the reduction of several specific urinary collagen fragments, most from collagen alpha-I(I). This decrease in collagen fragments may indicate reduced collagen degradation within the extracellular matrix, which is expected to result in increased fibrosis. Fibrosis has been associated with various diseases affecting different organs, including the liver, kidney, lungs, and heart [42]. Previous studies have demonstrated an association between fibrosis and poor outcome in patients with various pathologies [43–45]. Fibrosis may constitute the “first hit” and induce vulnerability to “second hit” events either in e.g., infectious or general (cardiovascular) scenarios. In this context, a pre-existing fibrotic condition may render an organ/ tissue more vulnerable to further damage or insults from a second event or trigger. Fibrosis alters the normal structure and function of the affected tissue, compromising its capacity to respond and recover from subsequent insults. Consequently, when a second hit, such as infection or inflammation occurs, it can lead to more severe complications and worsen the overall outcome.

The concordance of significant changes in individual peptides observed due to critical/lethal COVID-19 appears to be higher in the context of ICU than in non-ICU subjects. While an objective measure to assess significant differences does not seem to exist, a concordance (based on up- or down-regulation) of 97% (in the case of ICU) compared to 76% (in the case of non-ICU) is at least indicative.

As expected, there are similarities in changes in biomarkers in patients developing the critical condition, irrespective of the underlying pathology and disease aetiology. At the same time, it becomes evident that specific changes, a decrease of peptides from CD99 antigen, are associated more specifically with critical COVID-19, and cannot be consistently associated with all-cause death, neither in nor outside ICU. This suggests that the “second hit” in the context of a SARS-CoV-2 infection is depicted via peptides deregulated in severe COVID-19 only, like CD99 antigen. This study's findings align with prior research reporting an association of urinary peptides (or classifiers based on theme) with unfavourable outcome. A Pubmed search using the keywords (urine OR urinary) AND (peptidom* OR proteom*) AND (death OR mortality) in the title or abstract resulted in 96 publications. After a manual assessment by three authors, 11 manuscripts were found to be relevant. These studies explored the association of urinary peptides with mortality in humans and include those describing the development of COV50 [11, 12]. Currie et al. described a significant value of CKD273, a classifier based on 273 urinary peptides, in predicting mortality in 155 microalbuminuric type 2 diabetic patients [46]. Similar results were presented by Verbeke et al., linking CKD273 to mortality in 451 chronic kidney disease patients [36]. Nkuipou-Kenfack et al. reported an association between urinary peptides and death, developing a classifier to predict mortality after ICU stay in 1243 patients [38]. In 2021, Martens et al. described the connection between multiple urinary peptides, including many collagen fragments, biological age, and mortality [18]. Batra et al. presented a proteomics-based mortality signature in COVID-19 and acute respiratory distress syndrome patients [47]. In the context of hepatocellular carcinoma, Bannaga et al. identified several urine peptides being significantly associated with death [48]. Recently, Wei et al. reported on the detection of urinary peptides related to pulse-wave velocity also linked to mortality [49]. In a robust study involving 1170 patients that underwent cardiac surgery, Piedrafita and colleagues identified 204 urinary peptides associated with acute kidney injury [50]. A classifier based on these 204 peptides was validated in an independent cohort of 1569 ICU patients, demonstrating good performance and significant association with mortality.

Collagen peptides were consistently prominent biomarkers across many of these studies, with reduced abundance being associated with an increased risk of death, as also demonstrated by He and colleagues in the context of heart failure [25]. Data from large cohorts in ICU and subjects not in critical condition at the time of sampling indicate that urinary peptides and classifiers derived thereof hold significant predictive value for a patient-relevant endpoint: death. In line with previous studies, the prediction of death appears to predominantly rely on collagen fragments, potentially reflecting attenuation of collagen degradation, and consequently progressing fibrotic processes. Evidently, the COV50 classifier was not designed to predict death in the general population. Additionally, considering the observation in this study that several peptides contained in this classifier show opposite regulation on predicting critical COVID-19 or death from any cause, it is to be expected that a classifier designed exclusively for death prediction, based solely on peptides significantly associated with death, could be of substantial value in guiding death-preventing interventions. Such a classifier is likely to be based mainly on collagen fragments.

The study has limitations. It relies on previously generated datasets; however, the large number of datasets, the high number of endpoints assessed, and the very high significance level of the findings strongly support the generalizability of the results. In fact, a strength of the study is the inclusion of datasets from various studies, underscoring the robust basis for this assessment.

## Conclusions

Collectively, this study demonstrates that the urinary COV50 classifier is significantly associated with future death in both ICU and non-ICU patients, allowing for the identification of “vulnerable” subjects, irrespective of the underlying conditions. Further research is necessary to assess whether specific, personalized intervention guided by urinary collagen fragments can significantly improve outcomes, ultimately reducing the risk of future mortality.

### Supplementary Information

**Additional file 1. **Descriptive statistics for ICU and non-ICU subjects stratified according to the study type from which the patients were derived.

**Additional file 2. **Methodology. Information is provided regarding urine sample preparation, proteome analysis by CE-MS, data processing, and sequencing of the urinary peptides.

## Data Availability

Anonymised data and code used in conducting the analyses will be made available upon request directed to the corresponding author. Proposals will be reviewed and approved by the authors with scientific merit and feasibility as the criteria. After approval of a proposal, data can be shared via a secure online platform after signing a data access and confidentiality agreement. Data will be made available for a maximum of 5 years after a data sharing agreement has been signed.

## Abbreviations

CE-MS: Capillary electrophoresis coupled to mass spectrometry
ICU: Intensive care unit
BMI: Body mass index
eGFR: Estimated glomerular filtration rate
HR: Hazard ratios
CI: Confidence interval

## References

[1] Gallo Marin B, Aghagoli G, Lavine K, Yang L, Siff EJ, Chiang SS (2021). Predictors of COVID-19 severity: a literature review. Rev Med Virol.

[2] Hartog N, Faber W, Frisch A, Bauss J, Bupp CP, Rajasekaran S (2021). SARS-CoV-2 infection: molecular mechanisms of severe outcomes to suggest therapeutics. Expert Rev Proteomics.

[3] Hojyo S, Uchida M, Tanaka K, Hasebe R, Tanaka Y, Murakami M (2020). How COVID-19 induces cytokine storm with high mortality. Inflamm Regen.

[4] Li CX, Gao J, Zhang Z, Chen L, Li X, Zhou M (2022). Multiomics integration-based molecular characterizations of COVID-19. Brief Bioinform.

[5] Tay MZ, Poh CM, Renia L, MacAry PA, Ng LFP (2020). The trinity of COVID-19: immunity, inflammation and intervention. Nat Rev Immunol.

[6] Pape HC, Moore EE, McKinley T, Sauaia A (2022). Pathophysiology in patients with polytrauma. Injury.

[7] Moore FA, Moore EE (1995). Evolving concepts in the pathogenesis of postinjury multiple organ failure. Surg Clin North Am.

[8] Morris CF, Tahir M, Arshid S, Castro MS, Fontes W (2015). Reconciling the IPC and two-hit models: dissecting the underlying cellular and molecular mechanisms of two seemingly opposing frameworks. J Immunol Res.

[9] Velez JCQ, Caza T, Larsen CP (2020). COVAN is the new HIVAN: the re-emergence of collapsing glomerulopathy with COVID-19. Nat Rev Nephrol.

[10] Tiwari NR, Phatak S, Sharma VR, Agarwal SK (2021). COVID-19 and thrombotic microangiopathies. Thromb Res.

[11] Staessen JA, Wendt R, Yu YL, Kalbitz S, Thijs L, Siwy J (2022). Predictive performance and clinical application of COV50, a urinary proteomic biomarker in early COVID-19 infection: a prospective multicentre cohort study. Lancet Digit Health.

[12] Wendt R, Thijs L, Kalbitz S, Mischak H, Siwy J, Raad J (2021). A urinary peptidomic profile predicts outcome in SARS-CoV-2-infected patients. Eclin Med.

[13] Siwy J, Wendt R, Albalat A, He T, Mischak H, Mullen W (2021). CD99 and polymeric immunoglobulin receptor peptides deregulation in critical COVID-19: a potential link to molecular pathophysiology?. Proteomics.

[14] Gayat E, Cariou A, Deye N, Vieillard-Baron A, Jaber S, Damoisel C (2018). Determinants of long-term outcome in ICU survivors: results from the FROG-ICU study. Crit Care.

[15] Latosinska A, Siwy J, Mischak H, Frantzi M (2019). Peptidomics and proteomics based on CE-MS as a robust tool in clinical application: The past, the present, and the future. Electrophoresis.

[16] Rodriguez-Suarez E, Siwy J, Zurbig P, Mischak H (2014). Urine as a source for clinical proteome analysis: from discovery to clinical application. Biochim Biophys Acta.

[17] Mischak H, Kolch W, Aivaliotis M, Bouyssie D, Court M, Dihazi H (2010). Comprehensive human urine standards for comparability and standardization in clinical proteome analysis. Proteomics Clin Appl.

[18] Martens DS, Thijs L, Latosinska A, Trenson S, Siwy J, Zhang ZY (2021). Urinary peptidomic profiles to address age-related disabilities: a prospective population study. Lancet Healthy Longev.

[19] Mavrogeorgis E, Mischak H, Latosinska A, Siwy J, Jankowski V, Jankowski J (2021). Reproducibility evaluation of urinary peptide detection using CE-MS. Molecules.

[20] Mischak H, Vlahou A, Ioannidis JP (2013). Technical aspects and inter-laboratory variability in native peptide profiling: the CE-MS experience. Clin Biochem.

[21] Mebazaa A, Casadio MC, Azoulay E, Guidet B, Jaber S, Levy B (2015). Post-ICU discharge and outcome: rationale and methods of the The French and euRopean Outcome reGistry in intensive care units (FROG-ICU) observational study. BMC Anesthesiol.

[22] Alkhalaf A, Zurbig P, Bakker SJ, Bilo HJ, Cerna M, Fischer C (2010). Multicentric validation of proteomic biomarkers in urine specific for diabetic nephropathy. PLoS ONE.

[23] Delles C, Schiffer E, von Zur MC, Peter K, Rossing P, Parving HH (2010). Urinary proteomic diagnosis of coronary artery disease: identification and clinical validation in 623 individuals. J Hypertens.

[24] Frantzi M, van Kessel KE, Zwarthoff EC, Marquez M, Rava M, Malats N (2016). Development and validation of urine-based peptide biomarker panels for detecting bladder cancer in a multi-center study. Clin Cancer Res.

[25] He T, Melgarejo JD, Clark AL, Yu YL, Thijs L, Diez J (2021). Serum and urinary biomarkers of collagen type-I turnover predict prognosis in patients with heart failure. Clin Transl Med.

[26] He T, Mischak M, Clark AL, Campbell RT, Delles C, Diez J (2021). Urinary peptides in heart failure: a link to molecular pathophysiology. Eur J Heart Fail.

[27] Htun NM, Magliano DJ, Zhang ZY, Lyons J, Petit T, Nkuipou-Kenfack E (2017). Prediction of acute coronary syndromes by urinary proteome analysis. PLoS ONE.

[28] Huang QF, Trenson S, Zhang ZY, Yang WY, Van Aelst L, Nkuipou-Kenfack E (2017). Urinary proteomics in predicting heart transplantation outcomes (uPROPHET)-rationale and database description. PLoS ONE.

[29] Kuznetsova T, Mischak H, Mullen W, Staessen JA (2012). Urinary proteome analysis in hypertensive patients with left ventricular diastolic dysfunction. Eur Heart J.

[30] Lindhardt M, Persson F, Zurbig P, Stalmach A, Mischak H, de Zeeuw D (2017). Urinary proteomics predict onset of microalbuminuria in normoalbuminuric type 2 diabetic patients, a sub-study of the DIRECT-Protect 2 study. Nephrol Dial Transplant.

[31] Packham DK, Wolfe R, Reutens AT, Berl T, Heerspink HL, Rohde R (2012). Sulodexide fails to demonstrate renoprotection in overt type 2 diabetic nephropathy. J Am Soc Nephrol.

[32] Rossing K, Bosselmann HS, Gustafsson F, Zhang ZY, Gu YM, Kuznetsova T (2016). Urinary proteomics pilot study for biomarker discovery and diagnosis in heart failure with reduced ejection fraction. PLoS ONE.

[33] Rotbain Curovic V, Magalhaes P, He T, Hansen TW, Mischak H, Rossing P (2021). Urinary peptidome and diabetic retinopathy in the DIRECT-protect 1 and 2 trials. Diabet Med.

[34] Rudnicki M, Siwy J, Wendt R, Lipphardt M, Koziolek MJ, Maixnerova D (2021). Urine proteomics for prediction of disease progression in patients with IgA nephropathy. Nephrol Dial Transplant.

[35] Tofte N, Lindhardt M, Adamova K, Bakker SJL, Beige J, Beulens JWJ (2020). Early detection of diabetic kidney disease by urinary proteomics and subsequent intervention with spironolactone to delay progression (PRIORITY): a prospective observational study and embedded randomised placebo-controlled trial. Lancet Diabetes Endocrinol.

[36] Verbeke F, Siwy J, Van Biesen W, Mischak H, Pletinck A, Schepers E (2021). The urinary proteomics classifier chronic kidney disease 273 predicts cardiovascular outcome in patients with chronic kidney disease. Nephrol Dial Transplant.

[37] Zhang Z, Staessen JA, Thijs L, Gu Y, Liu Y, Jacobs L (2014). Left ventricular diastolic function in relation to the urinary proteome: a proof-of-concept study in a general population. Int J Cardiol.

[38] Nkuipou-Kenfack E, Latosinska A, Yang WY, Fournier MC, Blet A, Mujaj B (2020). A novel urinary biomarker predicts 1-year mortality after discharge from intensive care. Crit Care.

[39] Kalantari S, Jafari A, Moradpoor R, Ghasemi E, Khalkhal E (2015). Human urine proteomics: analytical techniques and clinical applications in renal diseases. Int J Proteomics.

[40] He T, Pejchinovski M, Mullen W, Beige J, Mischak H, Jankowski V (2021). Peptides in plasma, urine, and dialysate: toward unravelling renal peptide handling. Proteomics Clin Appl.

[41] Magalhaes P, Pontillo C, Pejchinovski M, Siwy J, Krochmal M, Makridakis M (2018). Comparison of urine and plasma peptidome indicates selectivity in renal peptide handling. Proteomics Clin Appl.

[42] Zhao X, Kwan JYY, Yip K, Liu PP, Liu FF (2020). Targeting metabolic dysregulation for fibrosis therapy. Nat Rev Drug Discov.

[43] Dweck MR, Joshi S, Murigu T, Alpendurada F, Jabbour A, Melina G (2011). Midwall fibrosis is an independent predictor of mortality in patients with aortic stenosis. J Am Coll Cardiol.

[44] Ekstedt M, Hagstrom H, Nasr P, Fredrikson M, Stal P, Kechagias S (2015). Fibrosis stage is the strongest predictor for disease-specific mortality in NAFLD after up to 33 years of follow-up. Hepatology.

[45] Gulati A, Jabbour A, Ismail TF, Guha K, Khwaja J, Raza S (2013). Association of fibrosis with mortality and sudden cardiac death in patients with nonischemic dilated cardiomyopathy. JAMA.

[46] Currie GE, von Scholten BJ, Mary S, Flores Guerrero JL, Lindhardt M, Reinhard H (2018). Urinary proteomics for prediction of mortality in patients with type 2 diabetes and microalbuminuria. Cardiovasc Diabetol.

[47] Batra R, Uni R, Akchurin OM, Alvarez-Mulett S, Gomez-Escobar LG, Patino E (2023). Urine-based multi-omic comparative analysis of COVID-19 and bacterial sepsis-induced ARDS. Mol Med.

[48] Bannaga A, Metzger J, Voigtlander T, Pejchinovski M, Frantzi M, Book T (2021). Pathophysiological implications of urinary peptides in hepatocellular carcinoma. Cancers.

[49] Wei D, Melgarejo JD, Thijs L, Temmerman X, Vanassche T, Van Aelst L (2022). Urinary proteomic profile of arterial stiffness is associated with mortality and cardiovascular outcomes. J Am Heart Assoc.

[50] Piedrafita A, Siwy J, Klein J, Akkari A, Amaya-Garrido A, Mebazaa A (2022). A universal predictive and mechanistic urinary peptide signature in acute kidney injury. Crit Care.

