# Supplementary material for: Urinary peptides provide information about the risk of mortality across a spectrum of diseases and scenarios
Source: J Transl Med. 2023 Sep 24;21:663. doi: 10.1186/s12967-023-04508-6 (PMC10518109; doi:10.1186/s12967-023-04508-6)
Supplement: Supplementary file 2 — Additional file 2. Methodology. Information is provided regarding urine sample preparation, proteome analysis by CE-MS, data processing, and sequencing of the urinary peptides. [file 12967_2023_4508_MOESM2_ESM.docx]

**Additional File 2: Methodology.** Information is provided regarding urine sample preparation, proteome analysis by CE-MS, data processing, and sequencing of the urinary peptides.

**METHODOLOGY**

**Urine sample preparation and proteome analysis by CE-MS**

Sample preparation and capillary electrophoresis coupled with mass spectrometry (CE-MS) analysis were performed as described previously [1-4]. Typically, the second mid-stream morning urine was investigated, and samples were stored below -20^o^C. The sample preparation involved the thawing of urine samples, followed by dilution through the combination of 700 μL of the sample with an equal volume of a solution containing 2 M urea, 10 mM NH_4_OH, and 0.02% sodium dodecyl sulfate (SDS). Subsequently, ultrafiltration of the samples was performed using a 20 kDa cut-off Centristat centrifugal filter device (Sartorius, Göttingen, Germany) to remove high molecular weight proteins. The resulting filtrate was then subjected to desalting with a PD-10 gel filtration column (GE Healthcare Bio Sciences, Uppsala, Sweden) equilibrated with 0.01% NH_4_OH in HPLC-grade water to eliminate urea, salts, and electrolytes. Following this, the samples were lyophilized, stored at 4°C, and later utilized for measurement via CE-MS.

Prior to the CE-MS analysis, samples were reconstituted in 10 μL of HPLC-grade H_2_O. The analysis was conducted using a P/ACE MDQ capillary electrophoresis system (Beckman Coulter, Fullerton, CA, USA) connected to a micro-TOF-MS (Bruker Daltonic, Bremen, Germany). The running buffer employed was composed of 20% acetonitrile (Sigma-Aldrich, Taufkirchen, Germany) in HPLC-grade water (Roth, Karlsruhe, Germany), supplemented with 0.94% formic acid (Sigma-Aldrich). The electrospray ionization interface provided by Agilent Technologies (Palo Alto, CA) was configured with a potential ranging from -4.0 to -4.5 kV. Spectra were recorded across a mass-to-charge ratio (m/z) spanning from 350 to 3000 and accumulated every 3 seconds.

**Data processing**

Following the CE-MS analysis, mass spectral ion peaks that represented identical molecules but at varying charge states were subjected to deconvolution into single masses using MosaFinder v1.4 software [5]. Only signals with charge states (z) greater than 1, observed in a minimum of 3 consecutive spectra, and exhibiting a signal-to-noise ratio of at least 4 were considered. As a result, a list of peaks that provided distinctive characteristics for each polypeptide, encompassing information about its mass and migration time, was generated. Data were calibrated utilising 3151 internal standards as reference data points for mass and migration time using global and local linear regression, respectively. As part of the calibration of signal intensity through linear regression, signals from 29 abundant peptides were used as internal standards [6]. Notably, this calibration procedure is reproducible and effectively addresses variations from both analytical and dilution factors. The derived peak list attributes each polypeptide with its calibrated molecular mass (Da), calibrated migration time (minutes), and normalized signal intensity.

**Sequencing**

Candidate biomarkers underwent sequencing using either CE-MS/MS or LC-MS/MS analysis, as described in [7]. For MS/MS experiments, an Ultimate 3000 nano-flow system (Dionex/LC Packings, USA) or a P/ACE MDQ capillary electrophoresis system (Beckman Coulter, Fullerton, CA) was employed, both coupled with a Q Exactive™ Plus Hybrid Quadrupole-Orbitrap™ Mass Spectrometer (ThermoFisher Scientific, Waltham, Massachusetts, USA). A data-dependent mode was employed. Survey full-scan MS spectra (from m/z 300 to 2000) were acquired in the Orbitrap. Sequentially, ions were isolated for fragmentation. Subsequently, RAW data files were subjected to searches against the UniProt [8] human database utilizing Proteome Discoverer 2.4 and the SEQUEST search engine [9]. Fixed modifications were absent, while variable modifications included the oxidation of methionine and proline. The minimum precursor mass was set to 790 Da, maximum precursor mass to 6000 Da, with a minimum peak count of 10. High-confidence peptides were established through parameters such as a cross-correlation (Xcorr) value above 1.9 and a rank of 1. Furthermore, a precursor mass tolerance of 5 ppm and a fragment mass tolerance of 0.05 Da were applied. To enhance the validity of derived peptides, the correlation between peptide charge at a pH of 2 and CE-migration time was exploited to minimize false-positive rates [10]. This strategy involved comparing the calculated CE-migration time of a sequence candidate based on its peptide sequence (specifically, the count of basic amino acids) with the experimental migration time.

**References**

1. Mischak H, Vlahou A, Ioannidis JP. Technical aspects and inter-laboratory variability in native peptide profiling: the CE-MS experience. Clin Biochem. 2013;46(6):432-43.

2. Staessen JA, Wendt R, Yu YL, Kalbitz S, Thijs L, Siwy J, et al. Predictive performance and clinical application of COV50, a urinary proteomic biomarker in early COVID-19 infection: a prospective multicentre cohort study. Lancet Digit Health. 2022;4(10):e727-e37.

3. Martens DS, Thijs L, Latosinska A, Trenson S, Siwy J, Zhang ZY, et al. Urinary peptidomic profiles to address age-related disabilities: a prospective population study. Lancet Healthy Longev. 2021;2(11):e690-e703.

4. Mavrogeorgis E, Mischak H, Latosinska A, Siwy J, Jankowski V, Jankowski J. Reproducibility Evaluation of Urinary Peptide Detection Using CE-MS. Molecules. 2021;26(23).

5. Latosinska A, Siwy J, Mischak H, Frantzi M. Peptidomics and proteomics based on CE-MS as a robust tool in clinical application: The past, the present, and the future. Electrophoresis. 2019;40(18-19):2294-308.

6. Jantos-Siwy J, Schiffer E, Brand K, Schumann G, Rossing K, Delles C, et al. Quantitative urinary proteome analysis for biomarker evaluation in chronic kidney disease. J Proteome Res. 2009;8(1):268-81.

7. Klein J, Papadopoulos T, Mischak H, Mullen W. Comparison of CE-MS/MS and LC-MS/MS sequencing demonstrates significant complementarity in natural peptide identification in human urine. Electrophoresis. 2014;35(7):1060-4.

8. UniProt C. UniProt: the Universal Protein Knowledgebase in 2023. Nucleic Acids Res. 2023;51(D1):D523-D31.

9. Eng JK, McCormack AL, Yates JR. An approach to correlate tandem mass spectral data of peptides with amino acid sequences in a protein database. J Am Soc Mass Spectrom. 1994;5(11):976-89.

10. Zurbig P, Renfrow MB, Schiffer E, Novak J, Walden M, Wittke S, et al. Biomarker discovery by CE-MS enables sequence analysis via MS/MS with platform-independent separation. Electrophoresis. 2006;27(11):2111-25.
